# Personalized Medical Nutrition Therapy and Physical Exercise: The Future of Diabesity Care

**DOI:** 10.1007/s13679-026-00688-6

**Published:** 2026-03-12

**Authors:** Ludovica Verde, Giuseppe Annunziata, Elisabetta Camajani, Maria Grazia Tarsitano, Silvia Savastano, Annamaria Colao, Giovanna Muscogiuri, Massimiliano Caprio, Luigi Barrea

**Affiliations:** 1 Centro Italiano Per La Cura E Il Benessere del Paziente Con Obesità (C.I.B.O), Unità Di Endocrinologia, Diabetologia E Andrologia, Dipartimento Di Medicina Clinica E Chirurgia, Università Degli Studi Di Napoli Federico II, Naples, Italy; 2 Division of Endocrinology, Department of Medicine, The University of Arizona College of Medicine, Tucson, AZ USA; 3 Dipartimento Di Psicologia E Scienze Della Salute, Università Telematica Pegaso, Via Porzio, Centro Direzionale, Isola F2, 80143 Naples, Italy; 4 Laboratory of Cardiovascular Endocrinology, IRCCS San Raffaele, Rome, Italy; 5 Department for the Promotion of Human Sciences and Quality of Life, San Raffaele Open University, Rome, Italy; 6 Dipartimento Di Medicina Clinica E Chirurgia, Unità Di Endocrinologia, Diabetologia Ed Andrologia, Università Degli Studi Di Napoli Federico II, Naples, Italy; 7 Cattedra Unesco “Educazione Alla Salute E Allo Sviluppo Sostenibile”, Università Degli Studi Di Napoli Federico II, Naples, Italy

**Keywords:** Diabesity, Obesity, Diabetes, Nutrition, Diet, Physical exercise, Medical nutrition therapy, Ketogenic therapy, VLEKT, Mediterranean diet

## Abstract

**Purpose of the Review:**

Diabesity, the coexistence of type 2 diabetes mellitus (T2DM) and obesity, represents one of the major global health challenges. This review aims to synthesize current evidence on personalized medical nutrition therapy (MNT) and structured physical exercise as cornerstones of diabesity management, with a particular focus on very-low-energy ketogenic therapy (VLEKT).

**Recent Findings:**

Conventional pharmacotherapies improve glycemic control and promote weight reduction but often fail to fully address the multifactorial pathophysiology of diabesity. MNT has demonstrated significant efficacy in improving glycemic regulation, reducing weight, and modulating cardiometabolic risk factors. Among dietary strategies, the Mediterranean diet provides sustainable benefits, while more intensive interventions such as low-energy diets and ketogenic diets can induce rapid and clinically meaningful improvements, with emerging evidence supporting favorable effects on gut microbiota and inflammation. Complementary lifestyle interventions, particularly structured exercise programs combining aerobic and resistance training, further enhance metabolic outcomes and may contribute to T2DM remission in selected patients. Integration of MNT with incretin-based therapies holds promise for optimizing efficacy while preserving nutritional adequacy and functional health.

**Summary:**

Effective management of diabesity requires a multidisciplinary, precision-based approach. Personalized MNT and structured exercise represent foundational strategies, while pharmacological therapies provide valuable adjuncts. Among available options, VLEKT stands out for its ability to target key mechanisms of diabesity, including insulin resistance, adiposity, and chronic inflammation. Future diabesity care will rely on integrating nutrition, physical exercise, and pharmacotherapy within individualized frameworks to achieve sustained metabolic control and improved quality of life.

## Introduction

Diabesity, the coexistence of type 2 diabetes mellitus (T2DM) and obesity, has emerged as one of the defining public health challenges of the twenty-first century [1]. Its pathogenesis involves a complex interplay of insulin resistance, β-cell dysfunction, chronic low-grade inflammation, and oxidative stress, all of which accelerate cardiometabolic and endocrine complications [1]. The global escalation of obesity has driven a parallel rise in T2DM, underscoring the urgent need for integrated strategies beyond pharmacological care. Understanding the shared pathophysiological mechanisms underlying diabesity is essential to translate mechanistic insights into effective preventive and therapeutic interventions [1].

Conventional therapies, including metformin, insulin, sodium–glucose cotransporter 2 inhibitors (SGLT2is), and glucagon-like peptide-1 receptor agonists (GLP-1RAs), have advanced glycemic management and, in the case of GLP-1RAs, facilitated weight loss [2]. Yet, pharmacotherapy alone rarely addresses the full metabolic burden of diabesity [2].

Within this framework, medical nutrition therapy (MNT) has emerged as a cornerstone of care, providing individualized and evidence-based dietary strategies tailored to the complex clinical needs of affected individuals [1, 3].

MNT is not a static or one-size-fits-all intervention but rather a dynamic and adaptive process requiring continuous patient engagement, regular follow-up, and clinical monitoring [4, 5]. Registered dietitians play a central role, developing personalized nutritional plans to optimize outcomes such as lowering glycated haemoglobin (HbA1c), inducing sustained weight loss, improving lipid profiles, and preserving renal and cardiovascular function [6]. In contrast to pharmacological treatments, MNT directly targets lifestyle determinants of diabesity, empowering patients with long-term behavioural skills for disease self-management [5, 6]. Importantly, evidence demonstrates that MNT achieves clinically meaningful reductions in HbA1c and body weight, positioning it as a first-line therapy in diabesity management [5, 6].

Weight reduction remains central, as excess adiposity exacerbates insulin resistance and accelerates β-cell failure [7]. Evidence indicates that weight loss of 5–15% improves glycaemic control, blood pressure, and lipid profiles, while losses exceeding 10–15% can induce partial or complete T2DM remission in selected patients [7]. Accordingly, MNT employs multiple dietary strategies adapted to patient preferences, cultural contexts, and long-term adherence [5, 6].

Among these, the Mediterranean diet has consistently demonstrated efficacy in improving glycaemic outcomes and supporting sustained weight reduction [8]. Its nutrient-dense profile, rich in fiber, monounsaturated fats, polyphenols, and minimally processed foods, confers anti-inflammatory and antioxidant effects, translating into improved insulin sensitivity, reductions in HbA1c, and cardiovascular risk attenuation [9]. Despite its heterogeneity across regions, the Mediterranean diet remains the most consistently beneficial whole-food approach [10].

For patients requiring more intensive interventions, low-energy diets (LEDs) and very-low-energy diets (VLEDs) offer structured caloric restriction and have demonstrated rapid weight loss of 10–15% or more, often inducing diabetes remission when implemented early [11, 12].

Another emerging option is the ketogenic diet, particularly very-low-energy ketogenic therapies (VLEKTs), which shift metabolism toward fat oxidation and ketone production [13]. In diabesity, these regimens improve HbA1c, promote significant fat loss while preserving lean mass, and may beneficially modulate gut microbiota [13, 14]. Additionally, VLEKTs have been shown to reduce systemic inflammation and oxidative stress, likely through decreased circulating glucose and insulin levels, modulation of inflammatory cytokines, and enhanced antioxidant capacity [15]. These effects collectively contribute to improved insulin sensitivity, endothelial function, and overall metabolic health [13, 14], positioning VLEKT as a particularly effective intervention for addressing the multifactorial pathophysiology of diabesity.

Finally, intermittent fasting (IF) and time-restricted eating (TRE) are gaining attention as circadian-based strategies to optimize metabolic outcomes [16]. Early clinical trials report benefits on weight, fasting glucose, and HbA1c comparable to continuous energy restriction, with potential additional effects on insulin resistance and fat distribution [16, 17].

This review synthesizes current evidence on personalized MNT and physical exercise as integral components of diabesity care, highlighting their potential to complement and ultimately reshape the pharmacological paradigm. Particular attention is to the VLEKT, alongside structured exercise interventions, highlighting their potential as adjuncts or alternatives to conventional pharmacological approaches.

### Pathophysiology and Clinical Presentation of Diabesity

The coexistence of obesity and T2DM has become so widespread that the term “diabesity” characterized the twin epidemics, and it is now widely used in research and clinical practice [1, 18]. This concept highlights the intertwined pathophysiological mechanisms linking excess adiposity with impaired glucose metabolism, and it underscores the need for integrated approaches to prevention, diagnosis, and treatment [1, 18]. To understand diabesity as a distinct clinical entity, it is necessary to consider obesity and diabetes features separately.

Body mass index (BMI), waist circumference, and body fat percentage are commonly used to quantify obesity, which is characterised by an excessive accumulation of adipose tissue [19]. In obesity, adipocytes become dysfunctional and expand (hypertrophy) due to hypoxia, endoplasmic reticulum stress, and macrophage infiltration, thereby causing chronic low-grade inflammation [20]. Visceral adiposity is particularly harmful as enlarged free fatty acids (FFAs) are released into the portal vein, impairing hepatic insulin sensitivity and promoting increased gluconeogenesis and very-low-density lipoprotein (VLDL) secretion. These alterations establish a systemic environment favouring insulin resistance [21]. This is also why obesity-related clinical features include components of metabolic syndrome (e.g., hypertension, dyslipidemia, and impaired fasting glucose) [21], and why different obesity phenotypes have been clinically defined, such as metabolically healthy obesity (MHO), characterized by preserved insulin sensitivity and subcutaneous fat predominance, versus metabolically unhealthy obesity (MUO), with prominent visceral adiposity and insulin resistance [22].

T2DM is a complex metabolic disorder characterized by a dual defect: insulin resistance in peripheral tissues and progressive pancreatic pancreatic β-cell dysfunction that leads to compensatory hyperinsulinemia [23]. Mechanisms like glucotoxicity, lipotoxicity, oxidative stress, mitochondrial dysfunction, and amyloid deposition contribute to impaired insulin secretion. As a result, patients transition from normoglycemia to prediabetes and ultimately to overt diabetes, characterized by chronic hyperglycaemia and its consequences. The classic symptoms of T2DM (e.g., polyuria, polydipsia, and polyphagia) are often subtle or absent in the early stages [23].

Excessive fat accumulation associated with obesity, especially central obesity, drives systemic insulin resistance, with metabolic syndrome exacerbating metabolic abnormalities [24]. Initially, pancreatic β-cells compensate through increased insulin production, but this adaptation fails as ectopic fat deposition in the liver, pancreas, and skeletal muscle further exacerbates insulin resistance and disrupts insulin signaling pathways. Lipotoxic intermediates (e.g., diacylglycerols and ceramides) interfere with insulin receptor phosphorylation and downstream signaling, worsening metabolic dysfunction. In addition, the pro-inflammatory state of obesity contributes directly to β-cell dysfunction and vascular complications. Altered adipokine secretion is also critical: reduced adiponectin impairs insulin sensitivity, while hyperleptinemia with leptin resistance perpetuates appetite dysregulation and metabolic imbalance [24]. In addition, visceral adiposity drives a bidirectional dysfunction of hypothalamic energy homeostasis through multiple neuroendocrine pathways: excessive visceral free fatty acids directly trigger mediobasal hypothalamic microglial activation and astrocytosis within days of overnutrition, initiating a neuroinflammatory cascade characterized by pro-inflammatory cytokine production and endoplasmic reticulum stress that disrupts leptin receptor signaling [25]. Concurrently, elevated circulating leptin paradoxically fails to suppress appetite, establishing central leptin resistance that elevates the homeostatic body weight set point and perpetuates hyperphagia. This impaired hypothalamic sensing of leptin and glucose further compromises the autonomic and neuroendocrine control of pancreatic β-cell function and hepatic glucose output, thereby creating a self-amplifying neuroendocrine-metabolic vicious cycle wherein visceral adiposity-induced hypothalamic gliosis and leptin resistance sustain both systemic insulin resistance and progressive pancreatic dysfunction [25]. Other contributing factors to diabesity are an altered gut microbiota, impaired autophagy, and mitochondrial dysfunction. Ultimately, diabesity represents a self-perpetuating cycle of obesity-induced insulin resistance and β-cell decline, leading to progressive metabolic deterioration and increased cardiometabolic risk [18, 24].

In summary, adiposity-driven inflammation and ectopic fat accumulation initiate systemic insulin resistance, which amplifies hyperglycaemia and increases β-cell metabolic demand; this triggers initial compensatory β-cell hyperfunction, but sustained glucolipotoxicity—mediated by excessive circulating free fatty acids, oxidative stress, and pro-inflammatory cytokines—progressively impairs glucose sensing and insulin secretion, culminating in β-cell exhaustion, mass loss, and overt pancreatic dysfunction, thereby establishing a vicious cycle wherein β-cell failure perpetuates hyperglycaemia and exacerbates peripheral insulin resistance [25].

The clinical features of diabesity reflect the combined manifestations of obesity and T2DM [1, 24]. Patients with diabesity often present a marked central (abdominal) obesity with increased waist circumference, hyperglycaemia, hypertension, and dyslipidemia (e.g., metabolic syndrome). Hepatic steatosis and nonalcoholic fatty liver disease (NAFLD) are frequent comorbidities, often progressing to nonalcoholic steatohepatitis (NASH). Cardiovascular complications occur earlier and with greater severity compared to obesity or diabetes alone. Subclinical atherosclerosis, endothelial dysfunction, and left ventricular hypertrophy are common even before overt events such as myocardial infarction or stroke. Diabesity also accelerates the development of chronic kidney disease. Beyond metabolic and cardiovascular manifestations, patients may suffer from reduced physical performance, chronic fatigue, musculoskeletal pain, and obstructive sleep apnea. Psychological distress, including anxiety and depression, is highly prevalent and can impair adherence to lifestyle and pharmacological interventions. Recognizing diabesity as a distinct entity underscores the urgency of integrated management strategies aimed at preventing, diagnosing, and treating this dual epidemic, which represents one of the most pressing public health challenges of the twenty-first century [1, 24].

### Endocrine-Metabolic Comorbidities Associated With Diabesity

The pivotal role of obesity as a major driver of metabolic dysfunction, with T2DM representing the most severe and clinically relevant consequence, has already been addressed. The coexistence of T2DM with obesity substantially increases the risk of cardiovascular disease and metabolic complications, such as dyslipidemia and metabolic-associated fatty liver disease (MAFLD), thereby contributing to the progression of end-organ damage, with a subsequent higher risk of morbidity and premature mortality [1].

However, hormonal dysregulation in diabesity extends well beyond metabolic dysregulation and insulin resistance. This is due to the complex interplay existing among adipose tissue, pancreas, liver, and skeletal muscle with the main endocrine axes (hypothalamic–pituitary, -thyroid, -adrenal, -gonadal) [1].

Adipose tissue is now recognized as an active endocrine organ, undergoing complex phenomena of hypertrophy, hyperplasia, hypoxia, and macrophage infiltration in conditions of overnutrition, leading to altered secretion of adipokines and cytokines [26]. Interestingly, while the increase in fat mass is paralleled by an increase in adipocyte number and size, only adipocyte size is reverted to normal conditions after weight loss, whereas adipocyte number is not decreased, thereby facilitating weight regain [26]. Therefore, adiponectin levels decline while leptin and resistin concentrations rise, fostering central leptin resistance and reducing satiety, finally promoting chronic inflammation [27]. Subsequently, these processes exacerbate insulin resistance and promote ectopic lipid deposition in crucial organs, particularly in the liver and in the pancreas, thereby contributing to β-cell lipotoxicity and mitochondrial stress, creating a vicious self-perpetuating feedback loop [28].

The dysregulation of the insulin–glucagon axis represents the most relevant endocrine hallmark of diabesity. Within pancreatic islets, insulin resistance induces a compensatory β-cell hyperactivity with subsequent hyperinsulinaemia, culminating in β-cell exhaustion, dedifferentiation [29], and apoptosis, finally leading to defective insulin secretion. On the other hand, concomitant α-cell dysfunction is responsible for inappropriately elevated glucagon production, even in the presence of hyperglycaemia, further fostering hepatic glucose output and hyperglicaemia [30].

Endothelial dysfunction represents a critical initiating event in atherosclerosis and serves as a sensitive indicator of vascular integrity [31]. Excess insulin levels and lipid overload play key roles in impairing the endothelium’s protective properties, thereby fostering conditions that promote atherosclerotic disease. A hard body of evidence clearly demonstrated that endothelial dysfunction is the common mechanism through which diabetes contributes to vascular disease and cardiovascular complications [31]: Chronic hyperglycaemia alters endothelial function through a reduction in nitric oxide bioavailability, an increase in oxidative stress, and the promotion of advanced glycation end-product formation, which further amplifies inflammation and inhibits nitric oxide synthesis [32]. In parallel, hyperinsulinemia, due to insulin resistance, further contributes to endothelial injury by disrupting insulin-mediated phosphatidylinositol 3-kinase/protein kinase B (PI3K/Akt) signaling and endothelial nitric oxide synthase phosphorylation, shifting signaling toward mitogen-activated protein kinase/extracellular signal–regulated kinase (MAPK/ERK) pathways and further compromising vascular homeostasis [32].

Endothelial dysfunction represents an initiating event in atherogenesis and is amplified by dyslipidemia, particularly through the actions of oxidized LDL [33]. OxLDL induces endothelial activation by impairing nitric oxide bioavailability, disrupting nitric oxide synthase signaling, and promoting the expression of proinflammatory cytokines and adhesion molecules [33].

It is well known that an altered activation of the hypothalamic–pituitary–adrenal (HPA) axis is strictly associated with obesity; in fact, a clear association between diurnal cortisol profile and markers of abdominal obesity, independently of demographics, socioeconomic status, medications, and smoking, has been demonstrated [34]. Disruption of the HPA axis determines altered circadian rhythm and reduced glucocorticoid receptor sensitivity, further enhancing visceral adiposity, increased gluconeogenesis, and insulin resistance [34], with important alterations of glucose metabolism [35]. Glucocorticoids are well-known to decrease insulin secretion, increase insulin resistance, and display negative effects on every component of the metabolic machinery controlling glucose homeostasis [36]. Interestingly, hypercortisolism represents a contributing cause in more than 10% of individuals with difficult-to-control T2DM, and the Catalyst multicenter study has been designed to assess the therapeutic potential of glucocorticoid receptor antagonism in this context [37]. On the other hand, diabesity profoundly disrupts both the hypothalamic–pituitary–adrenal (HPA) and hypothalamic–pituitary–gonadal (HPG) axes through metabolic and inflammatory stress [38]. Chronic hyperglicemia, insulin resistance, and adipose tissue dysfunction promote sustained activation of the HPA axis, leading to elevated cortisol levels that further worsen glucose dysregulation, central adiposity, and inflammation [39]. At the same time, excess adiposity alters the gonadal axis in males and females by disrupting gonadotropin-releasing hormone (GnRH) pulsatility and reducing pituitary luteinizing hormone (LH) and follicle-stimulating hormone (FSH) secretion [39] and by directly inhibiting testosterone production in males through excess leptin plasma levels [40]. Together, these alterations create a feed-forward loop in which endocrine dysfunction reinforces metabolic disease and cardiometabolic risk [41].

Subclinical thyroid dysfunction frequently coexists with diabetes, most often as elevated thyroid-stimulating hormone (TSH) with low-normal thyroxine (T4) plasma levels, leading to reduced basal metabolic rate, dyslipidaemia, and impaired glucose homeostasis, thereby worsening metabolic control and cardiovascular risk [42]. Thyroid hormones control hepatic gluconeogenesis, peripheral glucose uptake, and insulin sensitivity; therefore, even minor alterations of thyroid hormone production are able to worsen glycemic control. Notably, a recent meta-analysis showed that obesity is also significantly associated with an increased risk of overt and subclinical hypothyroidism, as well as Hashimoto thyroiditis [43], suggesting a bidirectional relationship between obesity and thyroid disease. Therefore, a careful evaluation of thyroid functions in individuals with obesity is mandatory, and prevention of obesity is crucial to successfully treating thyroid diseases.

Disorders of the reproductive axis, both in females and in males, are frequently observed in patients with diabetes. In women with diabetes, a wide spectrum of reproductive disorders and alterations of the HPG axis have been described, e.g., delayed puberty and menarche, menstrual cycle abnormalities, hypofertility and infertility, adverse pregnancy outcomes, and early menopause [44].

In men with T2DM, numerous studies have clearly demonstrated a relevant percentage of patients with low testosterone plasma concentrations, together with inappropriately low LH and FSH [45]. Notably, low plasma testosterone also predicts worsening of glucose control and increased mortality [46]. Obesity and insulin resistance suppress GnRH and LH secretion, resulting in hypogonadotropic hypogonadism, low testosterone, sarcopenia, and sexual dysfunction. On the other hand, increased leptin levels, which represent a hallmark of obesity, are known to directly suppress testosterone production by Leydig cells [40], and an inverse correlation between leptin levels, BMI, and testosterone secretion has been clearly demonstrated in men with obesity, with leptin representing the best hormonal predictor of the lower circulating androgen levels [47].

Interestingly, vitamin D deficiency, a frequent endocrine feature in diabesity, is known to impair β-cell function and reduce insulin sensitivity [48]. In fact, maintaining an adequate vitamin D status has been found effective in decreasing the risk of developing T2DM in subjects with prediabetes [49].

Mechanistically, all described endocrine derangements derive from common pathophysiological alterations characterizing diabesity, e.g., glucotoxicity, lipotoxicity, oxidative stress, and mitochondrial dysfunction [50]. Thus, endocrine abnormalities in diabesity do not represent secondary consequences but are main drivers of its progression. Clinically, these disturbances have major clinical implications: subtle thyroid dysfunction, HPA hyperactivity, hypogonadism, and vitamin D deficiency often remain undetected but are able to worsen metabolic control and can cause resistance to treatment, thereby increasing the risk for complications. Correcting endocrine imbalances can offer therapeutic leverage, improving glycaemic control and metabolic outcomes when integrated with lifestyle and pharmacologic interventions.

Overall, diabesity should be considered a complex endocrine disorder, encompassing adipose tissue inflammation and β-cell failure, together with alteration of the adrenal, thyroid, and gonadal axis. Recognizing and addressing these interconnected abnormalities may provide more effective strategies to halt the parallel epidemics of obesity and diabetes.

### Medical Nutrition Therapy in Diabesity

Effective management of diabesity requires an integrated therapeutic approach that simultaneously targets glycaemic control, weight management, and the prevention of obesity-related comorbidities [1, 3]. Within this framework, MNT emerges as a cornerstone of care, offering personalized and evidence-based dietary strategies tailored to the complex clinical needs of individuals living with both T2DM and obesity [1, 3].

MNT in diabesity is not a one-size-fits-all intervention but a dynamic process that requires ongoing patient engagement, regular follow-up, and adaptation to changing health circumstances [4, 5]. Registered dietitians play a central role in this process, designing individualized nutrition plans that align with therapeutic goals such as lowering HbA1c, achieving sustained weight reduction, improving lipid profiles, and preserving kidney and cardiovascular function [6]. In contrast to pharmacological treatments, MNT addresses the root lifestyle determinants of diabesity, enabling patients to acquire long-term behavioural skills necessary for disease self-management [4, 5]. Importantly, evidence consistently demonstrates that MNT can achieve clinically meaningful reductions in HbA1c and induce weight loss sufficient to improve insulin sensitivity and reduce cardiometabolic risk, positioning it as a first-line therapy for patients with diabesity [4, 5]. At the molecular level, these benefits are mediated through reductions in ectopic fat accumulation, attenuation of chronic low-grade inflammation, improved adipokine secretion, and decreased hepatic lipogenesis, all of which contribute to enhanced insulin signaling.

Weight reduction is particularly critical in the context of diabesity, as excess adiposity exacerbates insulin resistance and accelerates β-cell dysfunction [7]. Current evidence suggests that weight loss of 5–15% is associated with significant improvements in glycaemic control, blood pressure, and lipid parameters, while reductions exceeding 10–15% can even induce partial or complete remission of T2DM in selected individuals [7]. Thus, one of the primary objectives of MNT in diabesity is the induction and maintenance of clinically significant weight loss, achieved through sustainable dietary strategies that are compatible with patient preferences, cultural contexts, and long-term adherence.

Among the various dietary approaches, the Mediterranean diet has consistently demonstrated efficacy in improving both glycaemic and weight-related outcomes in individuals with diabesity [8]. Rich in whole grains, vegetables, fruits, legumes, nuts, and extra virgin olive oil (EVOO), this dietary pattern provides a nutrient-dense framework that supports both energy reduction and metabolic regulation [8]. For patients with diabesity, the Mediterranean diet exerts dual benefits: its high fiber and monounsaturated fat content improve insulin sensitivity and postprandial glucose excursions, while its emphasis on minimally processed, satiating foods facilitates spontaneous energy restriction and weight loss. These effects are partly driven by improved adipokine profiles, reduced oxidative stress, and modulation of inflammatory pathways involved in insulin resistance. Clinical studies have shown that adherence to the Mediterranean diet in patients with diabesity reduces HbA1c by up to 0.9%, lowers cardiovascular risk markers, and promotes moderate but sustained reductions in body weight [8]. Moreover, adopting the Mediterranean diet is strongly supported by its healthy components. Notably, hydroxycinnamic derivatives, quercetin, resveratrol, oleuropein, and hydroxytyrosol, which are well recognized for their antioxidant and anti-inflammatory activities, also exhibited anti-obesity functions [9]. These findings are in line with a recent comprehensive review, which emphasized the Mediterranean diet as the most consistently beneficial nutritional approach for improving both obesity- and diabetes-related outcomes in individuals with diabesity, while also acknowledging the potential of other dietary strategies [51]. Importantly, its flexibility and cultural adaptability make it one of the most sustainable long-term strategies for patients struggling with the dual burden of obesity and diabetes [51]. However, it is important to recognize that the Mediterranean diet is not a single, uniform dietary pattern but rather a heterogeneous collection of regional dietary traditions across the Mediterranean basin [10]. This variability can complicate comparisons across studies and raises the question of whether the observed benefits are unique to the Mediterranean diet or simply reflective of general principles common to many healthful whole-foods diets [10].

Nevertheless, while the Mediterranean diet offers a balanced, whole-foods framework, some patients with diabesity require more intensive interventions to achieve clinically meaningful weight loss. In this regard, LEDs and VLEDs have proven highly effective [12]. Typically delivered within structured clinical programs, these regimens provide significant caloric restriction, often through total diet replacement in the initial phases, followed by food reintroduction and long-term weight maintenance strategies [12]. In patients with obesity and with recently diagnosed T2DM, LEDs and VLEDs have been shown to induce rapid weight loss of 10–15% or more, frequently leading to T2DM remission when implemented early in the disease course [11]. From a mechanistic perspective, rapid reductions in visceral and hepatic fat improve hepatic insulin sensitivity and β-cell function, contributing to early glycaemic normalization. For patients with diabesity, such structured energy-restricted programs represent a powerful non-pharmacological tool to break the vicious cycle of hyperglycaemia and adiposity [11, 12]. However, their intensive nature requires medical oversight, careful monitoring of nutritional adequacy, and psychological support to maximize adherence and minimize dropout rates [11, 12].

Another nutritional strategy of growing interest in diabesity is the use of ketogenic diets [52, 53]. These regimens, by drastically reducing carbohydrate intake, shift metabolism towards fat oxidation and ketone body production, thereby lowering fasting and postprandial glucose levels [52, 53]. In patients with diabesity, ketogenic diets, particularly VLEKTs, have demonstrated significant improvements in both HbA1c and body weight, with some studies reporting T2DM remission following substantial weight reduction [52–54]. Notably, recent evidence suggests that VLEKTs may also help preserve lean body mass while promoting fat loss, supporting both metabolic and functional health [54]. Moreover, compared to a Mediterranean diet, VLEKTs appear to more strongly modulate the gut microbiota, with increases in beneficial bacterial taxa such as *Verrucomicrobia* and *Akkermansia* observed during the early months of intervention, which may contribute to improved metabolic outcomes [54]. These microbiota-driven effects may further influence adipose tissue inflammation and insulin sensitivity, reinforcing the metabolic benefits of carbohydrate restriction. The benefits for patients with diabesity are twofold: reduction of carbohydrate load directly improves glycaemic control, while concomitant energy restriction facilitates pronounced weight loss. However, VLEKTs are highly restrictive, require close medical supervision, and may pose risks such as nutrient deficiencies, gastrointestinal symptoms, and reduced long-term adherence [52, 53]. For patients with diabesity, ketogenic approaches may serve as effective short- to medium-term interventions but require careful patient selection and structured follow-up to ensure safety and sustainability.

In addition to macronutrient-focused approaches, intermittent fasting and TRE have gained popularity as innovative strategies for diabesity management [16, 17]. These regimens focus on meal timing rather than food composition, leveraging circadian biology to optimize metabolic outcomes [16, 17]. A recent randomized controlled trial comparing IF 16:8 and IF 14:10 protocols to a control diet in obese patients with T2DM demonstrated significant reductions in body weight, fasting blood glucose, and HbA1c, with the IF 16:8 protocol achieving the greatest weight loss [17]. Although current evidence suggests that intermittent fasting produces comparable HbA1c improvements to continuous energy restriction [55], emerging data indicate potential benefits in reducing insulin resistance, improving fat distribution, and enhancing weight loss in individuals with diabesity [16]. Such approaches may offer a flexible alternative to traditional dieting, particularly for those who find structured caloric restriction challenging. However, long-term evidence on adherence, sustainability, and safety remains limited, and these regimens should be considered adjunctive rather than primary strategies in MNT.

A critical dimension of MNT in diabesity is personalization [4, 5]. Given the heterogeneity of patient responses to dietary interventions, individualized care plans must consider not only clinical parameters such as BMI, HbA1c, and comorbidity profiles but also psychosocial and cultural factors influencing dietary behaviours. Emerging evidence suggests that inter-individual variability in gut microbiota composition, genetic background, and chrononutrition-related factors may significantly modulate metabolic responses to specific dietary interventions. Behavioural counselling, motivational interviewing, and the integration of digital health tools can enhance patient engagement, improve self-efficacy, and support long-term adherence. Importantly, MNT should not be viewed as an isolated intervention but as a central component of a multidisciplinary, holistic care model that integrates diet, physical activity, behavioural therapy, and pharmacotherapy [4, 5].

In conclusion, MNT represents the foundation of effective diabesity management, offering a personalized, evidence-based, and sustainable strategy for addressing the dual burden of T2DM and obesity (Table 1)**.** Through structured dietary interventions ranging from balanced whole-food patterns such as the Mediterranean diet to more intensive approaches such as LEDs, VLEDs, ketogenic diets, and intermittent fasting, MNT provides flexible tools to achieve clinically significant weight loss, improve glycaemic outcomes, and reduce the risk of long-term complications. Central to its success is the role of the dietitian, who ensures individualized tailoring, ongoing monitoring, and integration of patient preferences into care plans. As the global prevalence of diabesity continues to rise, the implementation of MNT as a core therapeutic strategy remains essential for improving both metabolic health and quality of life in affected individuals.

**Table 1:** Nutritional strategies for diabesity: mechanisms, evidence, patient suitability, and limitations

Nutritional approach	Mechanisms and benefits	Most suitable patients	Limitations
Mediterranean Diet (MD)	High in fiber, MUFA, and polyphenols; improves insulin sensitivity, gut microbiota, and antioxidant capacity. Evidence shows HbA1c reduction, lower CV risk, moderate weight loss, and anti-obesity effects of polyphenols (e.g., oleuropein, resveratrol)	Patients needing sustainable, balanced, culturally adaptable approach	Heterogeneous across regions; lack of studies combining MD with incretin therapies
Low-Energy Diets (LEDs)/Very-Low-Energy Diets (VLEDs)	Severe calorie restriction leads to rapid weight loss; effective in inducing T2DM remission, especially when implemented early. Evidence supports 10–15% weight reduction and improved metabolic control	Patients with obesity and recent T2DM diagnosis or requiring rapid metabolic improvement	Requires structured program, medical supervision, risk of nutritional inadequacy, high dropout risk
Very-Low-Energy Ketogenic Therapy (VLEKT)	Carbohydrate restriction induces ketosis, promoting fat oxidation and potentially preserving lean mass. Evidence shows improvements in HbA1c, body weight, and microbiota modulation (↑ Akkermansia, Verrucomicrobia) with greater short-term benefits vs MD	Patients needing pronounced weight loss and glycemic control; short-to-medium-term use	Restrictive, requires close follow-up; risk of GI symptoms, micronutrient deficiencies, adherence challenges
Intermittent Fasting/Time-Restricted Eating (TRE)	Focuses on meal timing, leveraging circadian biology. Evidence from RCTs shows comparable HbA1c reduction to CER and significant weight loss, with improvements in glucose and lipid profile	Patients struggling with daily calorie counting; those preferring flexible timing-based regimens	Long-term adherence and safety unclear; best as adjunctive strategy rather than primary

### Integrating Incretin-based Therapies Within MNT Frameworks

In recent years, the advent of novel antidiabetic agents has transformed the management of both diabetes and obesity [56]. Molecules such as GLP-1 receptor agonists and dual GIP/GLP-1 agonists, initially developed for glycemic control, have demonstrated significant effects on body weight reduction, offering new therapeutic opportunities for so-called diabesity. These agents provide an integrated approach whereby pharmacological treatment and MNT can be strategically combined to maximize metabolic benefits while preserving nutritional status and functional health [56].

Recent evidence underscores the potential benefits and risks of combining incretin-based therapies with specific nutritional strategies in patients with diabesity [56]. While agents such as semaglutide and tirzepatide amplify weight reduction and improve glycemic control, concerns about loss of lean body mass and micronutrient deficiencies highlight the need for cautious integration with restrictive dietary regimens [56]. VLEDs and LEDs may potentiate weight loss but carry a risk of lean mass loss and micronutrient deficiencies if protein and micronutrient intake are not carefully monitored. In contrast, preliminary evidence suggests that the VLEKT may help preserve lean body mass in patients with obesity, potentially mitigating some of these adverse effects. Among the various dietary strategies, the Mediterranean diet stands out as the most balanced and sustainable option [57]. Its high content of antioxidants, unsaturated fats, and dietary fiber supports incretin hormone response, promotes gut microbiota health, and is unlikely to exacerbate nutritional deficits [57]. While preliminary evidence highlights its safety and metabolic benefits, clinical studies specifically evaluating its combined use with incretin-based therapies are still lacking, and further research is warranted [57].

Preliminary data also suggest that targeted supplementation (e.g., berberine, α-lipoic acid) could further modulate metabolic pathways, although evidence on safety and long-term outcomes remains limited [4]. Within the framework of MNT, these findings reinforce the importance of tailoring dietary prescriptions to the pharmacological profile of each patient, ensuring that metabolic efficacy is achieved without compromising nutritional adequacy or functional health.

MD, Mediterranean Diet; MUFA, Monounsaturated Fatty Acids; CV, Cardiovascular; LED, Low-Energy Diet; VLED, Very-Low-Energy Diet; VLEKT, Very-Low-Energy Ketogenic Therapy; GI, Gastrointestinal; CER, Continuous Energy Restriction; IF, Intermittent Fasting; TRE, Time-Restricted Eating; RCT, Randomized Controlled Trial; T2DM, Type 2 Diabetes Mellitus.

### VLEKT and Diabesity

Among non-pharmacological interventions, the ketogenic diet, particularly its very low-calorie variant (VLCKD), represents an effective strategy for managing obesity and its metabolic complications [58]. Characterised by a daily carbohydrate intake of 30–50 g and a total energy intake below 800 kilocalories, VLCKD has been shown to induce rapid weight loss and is associated with additional metabolic benefits, including improved glycaemic control, reduced inflammation, and enhanced metabolic flexibility [59]. These properties make it particularly suitable for patients with diabesity, where both excess weight and insulin resistance coexist [18].

The metabolic mechanisms underlying VLCKD explain its clinical effects. Carbohydrate restriction lowers blood glucose and insulin while increasing glucagon, which promotes triglyceride mobilisation from adipocytes [60]. Free fatty acids are transported to the liver and converted into ketone bodies, which serve as alternative energy substrates for tissues such as muscle and brain. This state, termed “nutritional ketosis” (NK), differs from diabetic ketoacidosis (DKA) in ketone concentrations (0.5–3 mg/dL) and is accompanied by ketoadaptation, including enhanced enzymatic activity and mitochondrial density. Importantly, endogenous insulin preserves metabolic homeostasis and prevents DKA, supporting the safe application of VLCKD even in non–insulin-dependent diabetes [60].

To emphasise its therapeutic, medically supervised role, VLEKT is now referred to as “very low-energy ketogenic therapy” (VLEKT) [61]. VLEKT protocols proceed through staged phases: an initial ketogenic phase based on protein-rich meal replacements and low-carbohydrate vegetables, followed by gradual reintroduction of natural foods [60]. Protein intake is calibrated to 0.8–1.5 g/kg ideal body weight to prevent excessive gluconeogenesis, while fats are derived from high-quality unsaturated sources to sustain ketosis [60, 61]. Meal replacements provide precise macronutrient composition [60], simplify portion control [62, 63], improve adherence, reduce hedonic appetite [60, 64], preserve lean mass [65], stabilise insulin levels [58], and favourably influence gut microbiota [66].

Clinical outcomes align with these mechanisms. VLEKT is particularly effective in individuals with obesity and high insulin resistance, leading to improvements in body weight, fat mass, glycaemic control, and overall metabolic profile [67]. Its initial phase induces rapid weight loss through glycogen depletion and water excretion, which enhances patient motivation, though careful long-term management is required [68]. These characteristics underscore the concept of VLEKT as a medicalised nutritional intervention, analogous to pharmacotherapy, with benefits extending beyond mere weight reduction to broader metabolic health [69].

Despite its strong mechanistic rationale, clinical evidence for VLEKT in diabesity is limited. To date, only one longitudinal study has directly assessed its effects, comparing VLCKD with the Mediterranean diet in 11 patients with diabesity [54]. In this study, patients initially underwent a two-month VLCKD phase, followed by a gradual transition to a Mediterranean diet (VLCKD-MD), allowing comparison with a Mediterranean diet-only group over 12 months. VLCKD induced more pronounced improvements up to six months, including greater reductions in BMI (− 5.8 vs. − 1.7 kg/m^2^) and waist circumference (− 15.9 vs. − 5.2 cm), alongside significant improvements in HbA1c (− 1.2% vs. − 0.9%) and triglycerides (− 62.6 vs. − 13.2 mg/dL). These benefits were maintained relative to baseline and three-month outcomes. From a microbiological perspective, VLCKD-MD more favourably modulated gut microbiota composition, with a progressive increase in taxa linked to metabolic health up to month six, particularly the phylum *Verrucomicrobiota* and the genus *Akkermansia*. Although these markers decreased after six months, they remained above baseline levels [54].

While robust clinical trials are still lacking, the mechanistic rationale, metabolic effects, and preliminary evidence from longitudinal studies collectively support VLEKT as a highly promising nutritional approach for the management of diabesity. Its ability to improve anthropometric and metabolic parameters, modulate gut microbiota, and induce rapid, clinically relevant changes provides a strong foundation for its therapeutic use, highlighting the need for further research to confirm and refine its role.

The potential role of VLEKT in the nutritional management of diabesity is also addressed in the recent Consensus on Medical Nutrition Therapy in Diabesity (CoMeND), which, despite the lack of specific studies on this topic, highlights evidence supporting the endocrine–metabolic effects of this nutritional therapy and justifies its application in this pathological condition combining obesity and diabetes [70]. Notably, in providing dietary composition recommendations, the authors emphasize reducing carbohydrate intake (10–15% of daily calories), increasing protein intake (1 g/kg BW/day), and reducing saturated fat intake [70]. Taken together, these recommendations are consistent with the VLEKT approach, suggesting that VLEKT should be considered a temporary therapeutic option for acute management. In the long term, however, the maintenance nutritional approach should approximate these characteristics (though without necessarily inducing NK) by maintaining at least partial carbohydrate restriction, given the persistence of endocrine–metabolic alterations in these patients and their potential recurrence with the resumption of inappropriate eating habits.

### Role of Exercise in Diabesity

Physical activity, defined as any bodily movement that increases energy expenditure, and physical exercise, as a structured form of physical activity, are widely recognized as cornerstone strategies for lifestyle management in patients with diabesity [71].

In contrast, physical inactivity has been consistently associated with profound alterations in the expression, trafficking, and functional capacity of key genes and proteins regulating glucose homeostasis, whereas aerobic exercise exerts a stimulatory effect on these molecular pathways [72].

According to the European Association for the Study of Obesity Physical Activity Working Group, exercise plays a pivotal role in reducing body weight, improving insulin sensitivity, enhancing glycemic control, improving the cardiorespiratory system, and mitigating cardiovascular risk factors such as hypertension [73].

In a preclinical study, the impact of discontinuing and subsequently resuming high-intensity interval training (HIIT) was investigated in the context of diabesity [74]. A total of 75 C57BL/6 mice underwent five sequential stages: baseline, induction of diabesity with a Western diet, training, detraining, and retraining (each lasting six weeks). Following six weeks of HIIT, the trained groups exhibited elevated Vmax and maximum distance compared to their respective controls, indicating enhancements in cardiorespiratory fitness. Physical training also resulted in decreases in blood glucose levels and glycemic kinetics: in fact, when compared to its control group, HIIT reduced caloric intake by 24.5% and glycemic kinetics (GTT_AUC) by 18.6%, blood glucose by 7%, and increased Vmax by 13.1%. The authors concluded that detraining led to increased adiposity and deterioration of metabolic parameters and gut health, whereas retraining restored blood glucose regulation and improved intestinal health, although it did not reduce fat mass [74].

López-González et al. in a cross-sectional study of 24,224 Spanish workers (12,536 men and 11,688 women) assessed the association between diabesity and multiple determinants such as age, sex, socioeconomic status (SES), smoking, alcohol consumption, physical activity, adherence to the Mediterranean diet, and stress [75]. Their findings revealed that diabesity prevalence increases with advancing age, lower SES, tobacco or alcohol use, physical inactivity, poor adherence to the Mediterranean diet, and high stress levels [75].

In a systematic review and meta-analysis, Zhao and colleagues synthesized the available evidence on the effects of combined exercise (aerobic training, ≥ 150 min per week of moderate-to-vigorous intensity, and resistance training, at least two 60-min sessions per week, as recommended by national and international guidelines) on glycemic control, weight reduction, insulin sensitivity, blood pressure, and serum lipids in patients with T2DM and concurrent overweight/obesity [71]. Ten randomized controlled trials, including 978 participants, were analyzed. Pooled results demonstrated that combined exercise significantly reduced HbA1c, body mass index, homeostasis model assessment of insulin resistance, insulin and diastolic blood pressure [71].

Several studies have reported the possibility of T2DM remission in a subset of individuals through various interventions, including bariatric surgery and low-calorie diets, with or without substantial increases in physical activity [76]. Specifically, it has been observed that two years after a lifestyle intervention comprising structured exercise training (240–420 min per week distributed over 5 days), combined with an energy-restricted diet designed to achieve a 5–7% weight loss, approximately one in four individuals achieved remission of T2DM [77].

Taken together, regular physical activity—encompassing both aerobic and resistance training—improves mood and sleep quality, enhances muscle strength, and constitutes a powerful intervention to prevent the onset and progression of T2DM, while concurrently promoting body weight reduction. From a practical standpoint, and in line with current recommendations for individuals with T2DM, it is advisable to engage in physical activity with recovery intervals not exceeding 3 days, given that the effects on insulin sensitivity persist for approximately 24–72 h. Most clinical studies evaluating exercise in this population have adopted a frequency of three sessions per week, a schedule that many individuals also find easier to integrate into their daily routines [78].

A growing body of evidence supports the concept of precision exercise medicine, which is particularly relevant for the management of diabesity [79]. This approach acknowledges the substantial interindividual variability in responses to exercise, influenced by factors such as age, sex, baseline adiposity, cardiorespiratory fitness, and metabolic phenotype [80]. Statistical analyses highlight the heterogeneity of training responses, while work in molecular physiology and genomics has identified biological predictors of “trainability,” including genetic and transcriptomic signatures associated with changes in VO₂max and skeletal muscle adaptation [81, 82]. Collectively, these findings support the need for personalized exercise prescriptions tailored to individual metabolic characteristics to maximize benefits in glycemic regulation and cardiometabolic risk reduction [81, 82].

At the same time, strong evidence demonstrates that regular, sustained physical activity significantly reduces the progression from prediabetes to T2DM [83, 84]. The Diabetes Prevention Program showed that a lifestyle intervention emphasizing diet and ≥ 150 min per week of physical activity reduced diabetes incidence by 58% [84], with protective effects maintained over the long term, as confirmed in the 10-year follow-up [83].

## Conclusion

**Fig. 1:**
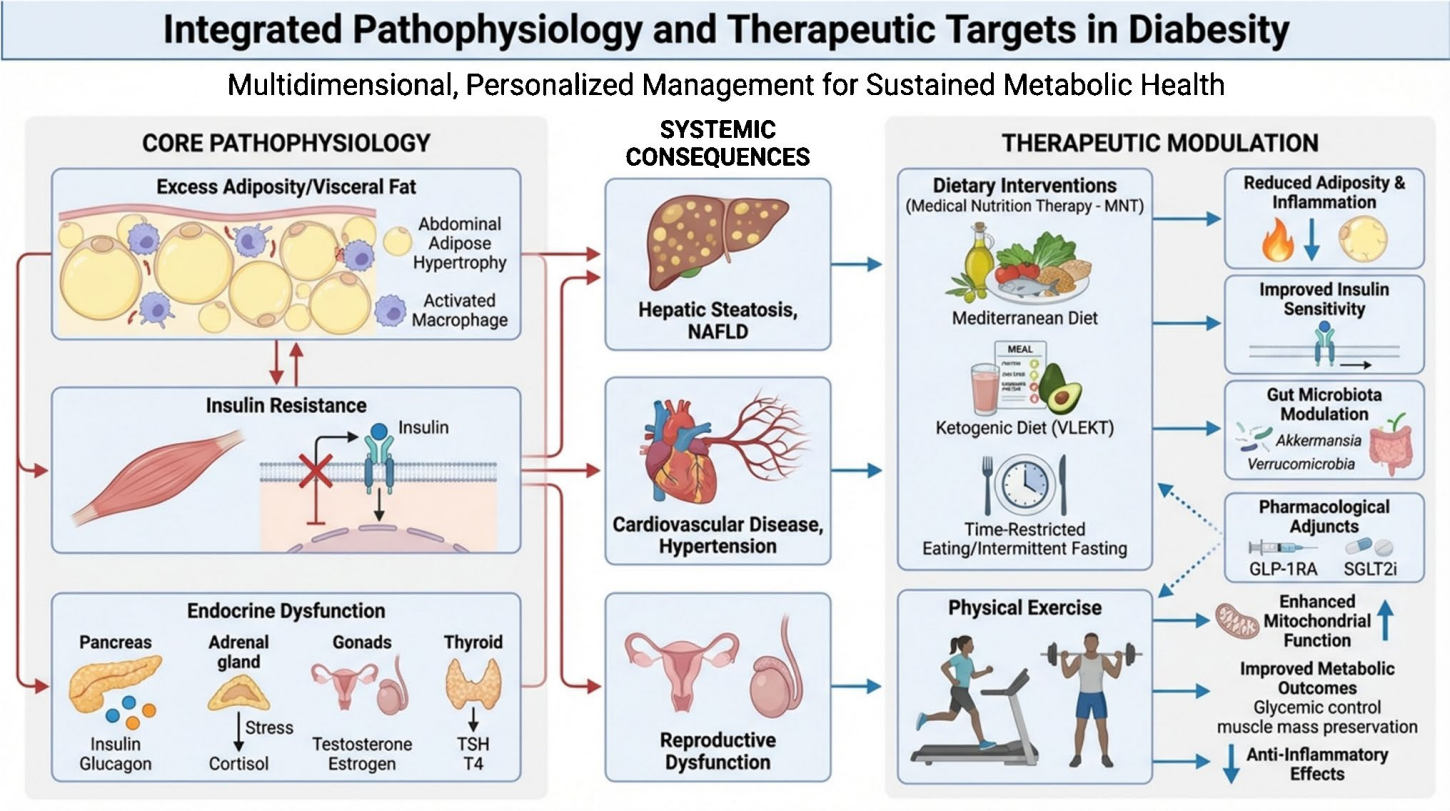
Pathophysiology and Integrated Management of Diabesity. Conceptual schematic illustrating the bidirectional interactions between excess adiposity, insulin resistance, and endocrine dysfunction underlying diabesity. The figure depicts the systemic consequences of these alterations on key target organs, including the liver, cardiovascular system, and reproductive axis. It also highlights the principal mechanistic pathways modulated by lifestyle-based interventions—specifically medical nutrition therapy and structured physical exercise—encompassing metabolic regulation, inflammatory signaling, and mitochondrial function. Dietary strategies (e.g., Mediterranean diet, low- and very-low-energy diets, very-low-energy ketogenic therapy, and intermittent fasting) and exercise interventions act synergistically to reduce visceral adiposity, improve insulin sensitivity, attenuate chronic low-grade inflammation, and enhance metabolic flexibility, thereby targeting the core drivers of diabesity and supporting long-term cardiometabolic health

The clinical management of diabesity demands a multidisciplinary strategy, where the complementary roles of nutritionists and physicians are essential to ensure durable outcomes. Nutritionists design, adapt, and supervise MNT, while physicians integrate these interventions with pharmacological care, comorbidity control, and long-term risk management. Within this integrated framework, the combination of targeted nutritional strategies and structured physical exercise plays a pivotal role in enhancing insulin sensitivity, reducing visceral adiposity, and attenuating chronic low-grade inflammation, thereby addressing key drivers of diabesity progression (Fig. 1).

Among available dietary strategies, VLEKT stands out for its ability to address core mechanisms of diabesity—namely insulin resistance, excess adiposity, and chronic inflammation. When delivered under medical supervision and incorporated into personalized MNT, VLEKT can achieve substantial improvements in glycemic regulation, weight reduction, and overall metabolic health. Importantly, when combined with tailored exercise programs, VLEKT may further potentiate metabolic benefits by preserving lean mass, improving mitochondrial function, and amplifying anti-inflammatory effects, thus supporting both metabolic and functional outcomes.

The future of diabesity care will rely on precision approaches that align nutritional therapies such as VLEKT with structured exercise programs and pharmacological advances. This integrated model targets the root drivers of the disease, supports sustained lifestyle change, and has the potential to redefine therapeutic standards while improving long-term quality of life. Looking forward, the implementation of multidimensional, personalized interventions—potentially informed by omics-based phenotyping, gut microbiota profiling, chrononutrition, and combined pharmacological–behavioral models—will be crucial to optimize treatment responsiveness and long-term disease remission. Further research is needed to identify patient subgroups most likely to benefit from specific integrative strategies and to translate mechanistic insights into scalable clinical practice.

## Key References

- Michaelidou, M., J.M. Pappachan, and M.S. Jeeyavudeen, Management of diabesity: Current concepts. World J Diabetes, 2023. 14(4): p. 396–411.
  - Diabesity, the coexistence of obesity and type 2 diabetes, represents a growing global health burden requiring integrated management. Effective treatment must address obesity-related complications and prioritise weight-neutral or weight-reducing antidiabetic agents. Comprehensive strategies combining pharmacological, lifestyle, and surgical interventions are essential for optimal metabolic and cardiovascular outcomes.
- Martemucci, G., et al., Comprehensive Strategies for Metabolic Syndrome: How Nutrition, Dietary Polyphenols, Physical Activity, and Lifestyle Modifications Address Diabesity, Cardiovascular Diseases, and Neurodegenerative Conditions. Metabolites, 2024. 14(6).
  - Metabolic syndrome serves as a pivotal link between diabesity, cardiovascular, and neurodegenerative disorders through metabolic and inflammatory dysregulation. Integrative interventions—emphasising polyphenol-rich diets, Mediterranean dietary patterns, microbiota modulation, and physical activity—offer promising strategies for prevention and metabolic improvement.
- Sindhwani, R., K.S. Bora, and S. Hazra, The dual challenge of diabesity: pathophysiology, management, and future directions. Naunyn Schmiedebergs Arch Pharmacol, 2025. 398(5): p. 4891–4912.
  - Diabesity, the coexistence of obesity and type 2 diabetes mellitus, arises from complex metabolic and inflammatory mechanisms driving cardiometabolic complications. Effective management requires integrated lifestyle, pharmacological, and surgical strategies tailored through a multidisciplinary and precision medicine approach.
- Pavlidou, E., et al., Diabesity and Dietary Interventions: Evaluating the Impact of Mediterranean Diet and Other Types of Diets on Obesity and Type 2 Diabetes Management. Nutrients, 2023. 16(1).
  - Recent clinical evidence highlights diet as a cornerstone in diabesity management, with the Mediterranean diet demonstrating superior metabolic and weight-related benefits. Personalised dietary strategies, integrating individual metabolic profiles, are essential to optimise long-term outcomes and curb the dual epidemic of obesity and diabetes.

## Data Availability

No datasets were generated or analysed during the current study.
